# TGR5 Expression Is Associated with Changes in the Heart and Urinary Bladder of Rats with Metabolic Syndrome

**DOI:** 10.3390/life11070695

**Published:** 2021-07-15

**Authors:** Chia-Chen Hsu, Kai-Chun Cheng, Yingxiao Li, Ping-Hao Hsu, Juei-Tang Cheng, Ho-Shan Niu

**Affiliations:** 1 Graduate Institute of Gerontology and Health Care Management, Chang Gung University of Science and Technology, Taoyuan City 33303, Taiwan; eardoctorhsu@yahoo.com.tw; 2 Department of Otorhinolaryngology, Taipei City Hospital, Taipei City 10341, Taiwan; 3 Department of Exercise and Health Sciences, University of Taipei, Taipei City 11153, Taiwan; 4 Department of Pharmacy, College of Pharmacy, Tajen University, Pingtung 90741, Taiwan; kc-cheng@tajen.edu.tw; 5 Pharmacological Department of Herbal Medicine, Department of Psychosomatic Internal Medicine, Kagoshima University Graduate School of Medical and Dental Sciences, Kagoshima 890-8544, Japan; 6 Department of Nursing, Tzu Chi University of Science and Technology, Hualien, 970302, Taiwan; sc145@ems.tcust.edu.tw; 7 School of Medicine, Chung Shan Medical University, Taichung City 40201, Taiwan; andy30817@gmail.com; 8 Department of Medical Research, Chi-Mei Medical Center, Tainan City 71004, Taiwan; jtcheng5503@gmail.com

**Keywords:** metabolic syndrome, TGR5, etanercept, heart, urinary bladder, rats

## Abstract

Adipose-derived cytokines may contribute to the inflammation that occurs in metabolic syndrome (MetS). The Takeda G protein-coupled receptor (TGR5) regulates energy expenditure and affects the production of pro-inflammatory biomarkers in metabolic diseases. Etanercept, which acts as a tumor necrosis factor (TNF)-α antagonist, can also block the inflammatory response. Therefore, the interaction between TNF-α and TGR5 expression was investigated in rats with high-fat diet (HFD)-induced obesity. Heart tissues isolated from the HFD-induced MetS rats were analyzed. Changes in TGR5 expression were investigated with lithocholic acid (LCA) as the agonist. Betulinic acid (BA) was used to activate TGR5 in urinary bladders. LCA was more effective in the heart tissues of HFD-fed rats, although etanercept alleviated the function of LCA. STAT3 activation and higher TGR5 expression were observed in the heart tissues collected from HFD-fed rats. Thus, cardiac TGR5 expression is promoted by HFD through STAT3 activation in rats. Moreover, the urinary bladders of female rats fed a HFD showed a low response, which was reversed by etanercept. Relaxation by BA in the bladders was more marked in HFD-fed rats. The high TGR5 expression in HFD-fed rats was characterized using a mRNA assay, and the increased cAMP levels were found to be stimulated by BA in the isolated bladders. Therefore, TGR5 expression increases with a HFD in both the hearts and urinary bladders. Collectively, cytokine-medicated TGR5 activation was observed in the hearts and urinary bladders of rats.

## 1. Introduction

Takeda G protein-coupled receptor (TGR5) belongs to the G protein-coupled receptor (GPCR) superfamily [1]. In addition to the heart [2], TGR5 is expressed in other organs and is amenable to being targeted by bile acids in both healthy and diseased states [3]. TGR5 is a metabolic regulator, which is also involved in inflammatory responses [4]. TGR5 activation induces cytoprotective changes in the heart [5,6]. At toxic concentrations, bile acid may stimulate cholinergic M2 receptors, which cause negative effects on myocardial contractility and heart rate [7]. Therefore, TGR5 activation is introduced to provide benefits to cardiac function [8]. Recently, it has been documented that cardiac TGR5 expression is promoted in type-1 diabetic rats [9], mainly due to hyperglycemia, which seems to be related to compensatory homeostasis. However, TGR5 expression in other metabolic disorders remains unknown.

Metabolic syndrome (MetS) belongs to a pre-diabetic state and is a prevalent, multifactorial, and complex disorder, associated with a higher risk of developing diabetes and other cardiovascular complications [10]. Management of MetS is required owing to an increase in the global prevalence [11]. Therefore, several animal models mimicking MetS have been developed, and a high-fat diet (HFD) is popularly used to feed animals [12]. Lipid-induced injury, known as lipotoxicity, is mainly associated with hyperlipidemia, a condition caused by a HFD [13]. Chronic inflammation is known to be linked to hyperlipidemia, causing the induction of cardiovascular diseases (CVDs) [14], because inflammation due to lipid accumulation and excess lipids may have an effect on cell membranes [15]. Therefore, the inflammation caused by a HFD is associated with metabolic disorders that have been observed in rats [12] and mice [16]. From animal studies, a HFD is known to trigger acute and/or chronic inflammation through a complex mechanism [17], with inflammation being mentioned as an important factor for changes in cardiac TGR5 expression [6].

Generally, inflammation is associated with an increase in the levels of plasma cytokines, including tumor necrosis factor alpha (TNF-α), interleukin-6 (IL-6), and interleukin-1 beta (IL-1β) [18]. These inflammatory markers have been widely identified in HFD-fed animals, such as cardiac inflammation observed in HFD-fed animals [19], with a higher expression of TNF-α [20]. TNF-α is known to be involved in cardiac injury, acting through inflammatory pathways and/or the activation of cell death programming, including apoptosis [21]. In a clinical setting, TNF-α inhibitors are widely used to treat TNF-α-associated disorders, including rheumatoid arthritis, psoriasis, ankylosing spondylitis, Crohn’s disease, diabetes mellitus, Alzheimer’s disease, and cancer [22]. These drugs, named anti-TNF agents, are mostly prepared from monoclonal antibodies, such as adalimumab, golimumab, infliximab, and certolizumab pegol, except etanercept, which is a receptor fusion protein [23]. They can suppress responses to TNF-α regardless of the receptor subtype [23]. In animal research, etanercept (Enbrel@), a TNF-α inhibitor [23], can block the activity of TNF-α competitively at an effective dose [23]. Moreover, in addition to heart failure, the overexpression of TNF-α in mice promotes the occurrence of cardiac hypertrophy [24]. It has been documented that etanercept may alleviate cardiac hypertrophy through inhibition of the TNF-α receptor [25]. Therefore, we were interested in determining the effect of etanercept on TGR5 expression in animals fed a HFD.

Bile acids are known to be involved in the regulation of the motility of organs, and lithocholic acid (LCA), the agonist of TGR5, has been demonstrated to relax the detrusor contractility of the urinary bladder in normal rats without TGR5 activation [26]. The specific TGR5 receptor agonist INT-777 has also been shown to fail to modify urinary contraction, although TGR5 expression was reported in the same research [26]. It seems that TGR5 expression in the urinary bladder is less sensitive in normal rats. Therefore, we were also interested in elucidating the changes in TGR5 expression in the urinary bladder using rats fed a HFD and those that were not.

Thus, in the present study, we investigated the changes in TGR5 expression in rats fed an HFD, with a focus on the urinary bladder and heart. Additionally, the potential role(s) of TNF-α and the possible mediation of the signal transducer and the activator of transcription 3 (STAT3) in changes in TGR5 expression were also determined in this study.

## 2. Materials and Methods

### 2.1. Animals

Sprague Dawley (SD) rats of either sex, weighing 250–270 g, were purchased from the National Laboratory Animal Center (Taipei, Taiwan). The animal experiments were approved (107101701) by the Institutional Animal Ethics Committee of Chi-Mei Medical Center. The animal experiments were performed in accordance with the Guide for the Care and Use of Laboratory Animals of the National Institutes of Health, as well as the guidelines of the Animal Welfare Act. Two sets of protocol were used, wherein male rats were assessed for cardiac performance and female rats received catheter insertion from the urethra into the bladder for obtaining a cystometrogram. For each experiment, the rats were divided into three groups (*n* = 12 in each group): (i) Rats fed normal chow as the normal control; (ii) HFD-fed rats treated with vehicle as the model control; and (iii) etanercept-treated HFD-fed rats. HFD-fed rats were allowed ad libitum intake of a diet containing 60% fat (wt/wt) (#58Y1; TestDiet, Richmond, IN, USA). The control group rats were fed standard rat chow (5% fat, #5001; LabDiet, St. Louis, MO, USA). After 6 weeks, the body weight of the rats was measured to confirm obesity: The HFD-induced group was markedly different from the normal group (*p* < 0.05). In the etanercept treatment group, 0.8 mg/kg of etanercept (Enbrel@; Wyeth Europa, Maidenhead, UK) was subcutaneously administered per week, divided into six or seven equal daily injections [27] for 4 weeks. Vehicle treatment was performed in the same manner as that performed with sterile water, the solvent for etanercept. After the end of treatment, the fasting rats were anesthetized with 2% isoflurane, and blood samples were collected from the femoral artery of eight rats in each group. Under 4% isoflurane, the male rats were then sacrificed. The hearts of rats were rapidly excised and rinsed by immersion in ice-cold Krebs–Henseleit buffer (KHB) for the Langendorff assay. Plasma prepared from whole blood after centrifugation was stored at –80 °C until the analyses. The heart tissues of the other male rats (*n* = 4) in each group were dissected immediately, washed with ice-cold saline, dried, and weighed. The isolated tissues were stored at −80 °C until further analysis.

### 2.2. Measurement of Blood Biomarkers

The levels of plasma pro-inflammatory cytokines TNF-α and IL-6 were estimated using enzyme-linked immunosorbent assay kits (Sigma-Aldrich, St. Louis, MO, USA). The plasma biomarkers of hepatic function, including aspartate aminotransferase (AST) and alanine aminotransferase (ALT), were evaluated using commercial kits (BioVision, Milpitas, CA, USA), according to the manufacturer’s protocol.

### 2.3. Cardiac Performance in Langendorff Apparatus

Measurements of cardiac performance were carried out using our previous method [28]. The rats were sacrificed under anesthesia induction with 3% isoflurane, and their hearts were excised rapidly and rinsed by immersion in ice-cold KHB. The hearts were mounted in the Langendorff apparatus and continuously perfused with warm (37 °C) and oxygenated (5% CO_2_ in O_2_) KHB at a constant pressure of 70 mmHg. The organ chamber temperature was maintained at 37 °C during the experiment. A water-filled latex balloon was inserted through an incision made in the left atrium into the left ventricle through the mitral valve and adjusted to a left ventricular end-diastolic pressure (LVEDP) of 5–7 mmHg during the initial equilibrium. The distal end of the catheter was connected to an iWorx 214 TM data acquisition system (LabScribe 2.0 software, iWorx Systems, Inc., Dover, NH, USA) through a pressure transducer for continuous recording. In each experiment, after allowing stabilization for 30 min through perfusion, the test agents were added to the KHB for further analysis. The female rats in another set received the same treatment as that of the male rats described above, and eight rats from each of the three groups were used for studying the changes in the urinary bladder using a cystometrogram, as described below.

### 2.4. Cystometrogram of Urinary Bladder

After the bladders were emptied in anesthetized rats, a urethral catheter was placed to fill the bladder, and saline was infused at a steady rate (0.08 mL/min) to measure the bladder pressure, as described previously [29]. Pressure and force signals were detected by connecting to an iWorx 214 TM data acquisition system, as mentioned above, through a pressure transducer for continuous recording. The cystometrogram parameters, including peak micturition pressure and duration of contractions, were recorded according to a previous method [30]. The peak micturition pressure was defined as the maximum pressure (cmH_2_O), and duration was defined as the time (s) of the intervals during micturition. Betulinic acid (Sigma-Aldrich, St. Louis, MO, USA) at a dose of 50 mg/kg administered through an intraperitoneal injection (ip) was identified to be effective on pressure and duration in preliminary experiments; it was then applied for comparing the parameters in the three groups. The activity of betulinic acid was calculated as the ratio (%) of decreased micturition pressure over non-treated pressure for performing a comparison among the three groups.

The urinary bladders in another four female rats from each group were isolated under 4% isoflurane, and the isolated fresh tissues were used for the measurement of cAMP, as described below. The other tissues were washed in ice-cold saline and stored at −20 °C until further analysis.

### 2.5. Measurement of Intracellular cAMP Levels in Isolated Urinary Bladders

Urinary bladder tissues were incubated with phosphodiesterase inhibitors (IBMX 5 µM, Sigma-Aldrich, St. Louis, MO, USA) for 30 min and treated with betulinic acid (5 µM) for another 1 h. Sample lysates were collected, and intracellular cAMP levels were measured using a cAMP Assay Kit (Abcam, Cambridge, MA, USA). Differences between treatment with betulinic acid or no treatment were indicated as the cAMP levels increased in each group.

### 2.6. Real-Time Quantitative PCR

According to our previous report [31], the mRNA levels of the signal transducer were determined. In brief, total RNA was extracted using TRIzol reagent (Thermo Fisher, Carlsbad, CA, USA) from cell lysates. Total RNA (200 ng) was reverse-transcribed into cDNA with random hexamer primers (Roche Diagnostics GmbH, Mannheim, Germany). PCR experiments were performed using a LightCycler (Roche Diagnostics GmbH, Mannheim, Germany). The concentration of each product was calculated from a corresponding standard curve. The relative gene expression was subsequently indicated as the ratio of the target gene level to that of β-actin. The primers for each factor were as follows:

TGR5
F: 5′-TGGCTGCTGTGACTCTTTGA-3′
R: 5′-TGTGACATCATGGGTCTTGG-3′
BNP
F: 5′-GTCAGTCGCTTGGGCTGT-3′;
R: 5′-CCAGAGCTGGGGAAAGAAG-3′;
β-MHC
F: 5′-CATCCCCAATGAGACGAAGT-3′;
R: 5′-GGGAAGCCCTTCCTACAGAT-3′;
β-actin
F: 5′-CTAAGGCCAACCGTGAAAAG-3′;
R: 5′-GCCTGGATGGCTACGTACA-3′

### 2.7. Western Blotting Analysis

Ice-cold radio-immuno-precipitation assay (RIPA) buffer was applied to extract the proteins from tissue homogenates, either from the urinary bladder or heart. Western blotting analysis was subsequently performed using our previous method [31]. The target antigens from the protein extracts were characterized using the primary antibodies specific for p-STAT3 (1:1000, phosphor-Y705, ab76315, Abcam), STAT3 (1:1000, ab68153, Abcam), TGR5 (1:1000, ab72608, Abcam), or β-actin (1:5000, A5316, Sigma-Aldrich, St. Louis, MO, USA). The bound primary antibodies were then hybridized to horseradish peroxidase-conjugated goat anti-rabbit or anti-mouse IgGs (Calbiochem, San Diego, CA, USA), and the immunoreactive bands were developed with a chemiluminescence kit (Perkin Elmer, Waltham, MA, USA). Based on the optical densities of each band, p-STAT3 (88 kDa), STAT3 (88 kDa), TGR5 (35 kDa), or β-actin (43 kDa) was quantified using our previous method [31].

### 2.8. Statistical Analysis

The results are indicated as the mean ± SEM of each group. The results were analyzed by two-way analysis of variance, followed by Dunnett’s post-hoc analysis, using SPSS analysis software (SPSS Inc., Chicago, IL, USA). A *p*-value of <0.05 was considered statistically significant.

## 3. Results

### 3.1. Role of TNFα in MetS Induced in HFD-Fed Rats

**Figure 1:**
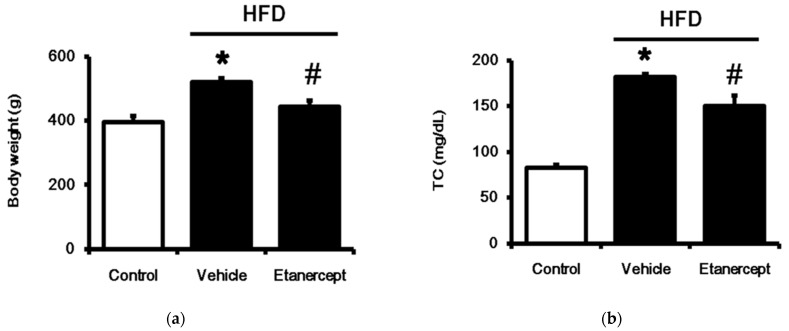

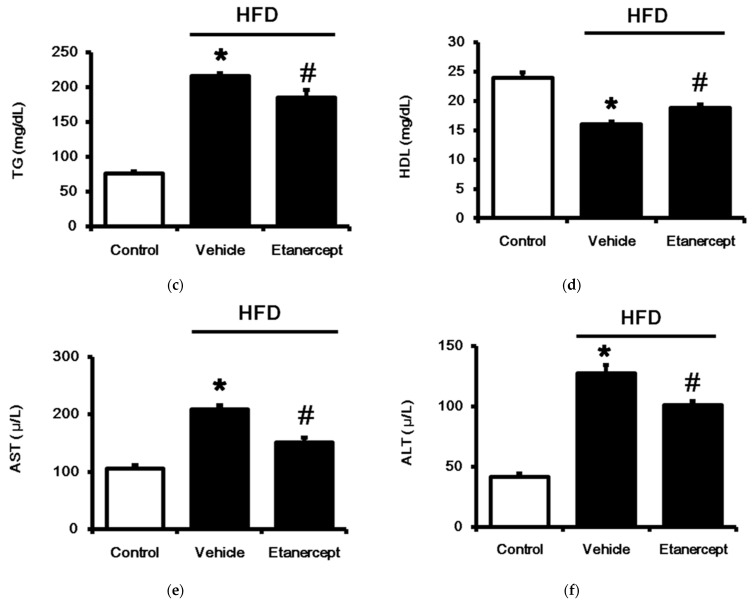
Identification of metabolic syndrome (MetS) in rats fed a high-fat diet (HFD). The rats were divided into three groups: (i) rats fed normal chow (control); (ii) HFD-fed rats treated with vehicle (HFD); (iii) etanercept-treated rats that received HFD (HFD + etanercept). They were compared in terms of body weight (**a**), plasma lipids, including total cholesterol (**b**), triglyceride (**c**), and high-density lipoprotein (HDL)-cholesterol (**d**), in addition to hepatic indicators, such as aspartate aminotransferase (AST) (**e**) and alanine aminotransaminase (ALT) (**f**) in plasma. The results in each column are shown as the mean ± SEM (*n* = 8 per group). * *p* < 0.05 vs. the control group; # *p* < 0.05 vs. the HFD group.

Rats fed a 60% HFD for 6 weeks were compared with rats that received normal chow. As shown in Figure 1, the body weight was significantly increased in the HFD-fed rats. Additionally, the plasma lipids, including total cholesterol and triglyceride, were also increased in the HFD-fed rats. However, the plasma high-density lipoprotein cholesterol level was reduced in the HFD-fed rats; otherwise, the levels of biomarkers of hepatic function, including AST and ALT, were higher in the HFD-fed rats. These changes were widely observed in the rats with MetS. Etanercept (Enbrel) was then administered to block TNF-α activity, as described previously [27], in another group of HFD-fed rats. The changes in metabolic disorders were markedly alleviated by etanercept, as shown in Figure 1. Additionally, MetS was also confirmed in the female rats fed a HFD in the same manner. Similarly, blockade of TNF-α using etanercept (Enbrel) reversed the changes in the HFD-fed female rats. Therefore, they were used to assay the functions of the urinary bladder.

### 3.2. Changes in Cardiac Performance in HFD-Fed Rats

**Figure 2:**
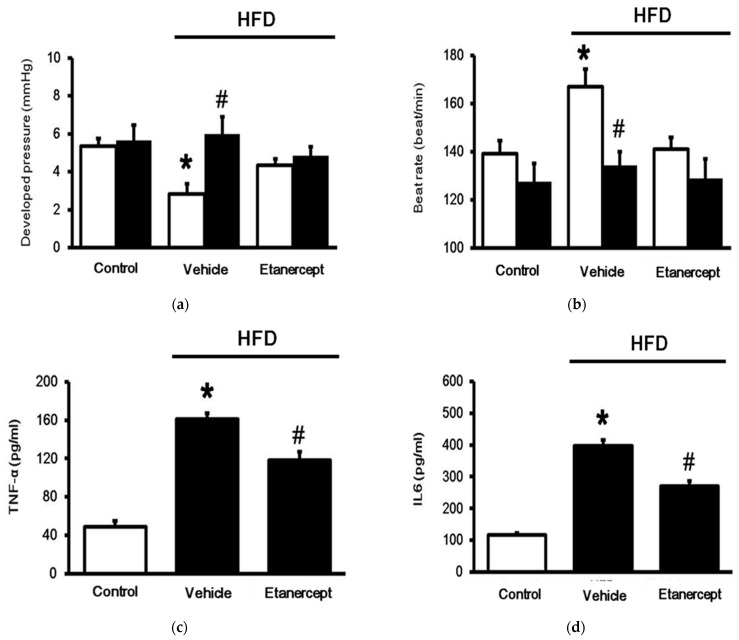
Changes in cardiac performance in isolated rat hearts were evaluated using the Langendorff apparatus. Cardiac contractility increased in the Langendorff-perfused heart in comparison with (**a**) and in addition to the changes in beat rate (**b**). The hearts were isolated from rats of three groups: (i) rats fed normal chow (control); (ii) HFD-fed rats treated with vehicle (HFD); (iii) etanercept-treated rats that received HFD (HFD + etanercept). The effect of 5 μM of LCA shown in the block column was compared with that in the open column, indicating the vehicle-treated control. Additionally, levels of plasma cytokines, including tumor necrosis factor (TNF)-α (**c**) and interleukin (IL)-6 (**d**), in each group were also compared. The results in each column are shown as the mean ± SEM (*n* = 8 per group). * *p* < 0.05 vs. the control group; # *p* < 0.05 vs. the HFD group.

Spontaneous contractility in the Langendroff apparatus was markedly reduced in the hearts of the HFD-fed rats compared to that in the hearts of the normal rats. LCA (Sigma-Aldrich, St. Louis, MO, USA) stimulated contractile responses (Figure 2a) and attenuated beating rates (Figure 2b) in the hearts isolated from the normal rats. Notably, the effect of LCA on cardiac performance was more significant in the hearts isolated from the HFD-fed rats than in those isolated from the control rats. However, the cardiac performance in the hearts isolated from the HFD-fed rats treated with etanercept exhibited less response than that in the hearts of the vehicle-treated group. The levels of plasma cytokines, including TNF-α (Figure 2c) and IL-6 (Figure 2d), were also increased in the HFD-fed rats compared to those in the normal rats. This effect was also reduced in the HFD-fed rats treated with etanercept.

### 3.3. Changes in TGR5 Expression in the Hearts

**Figure 3:**
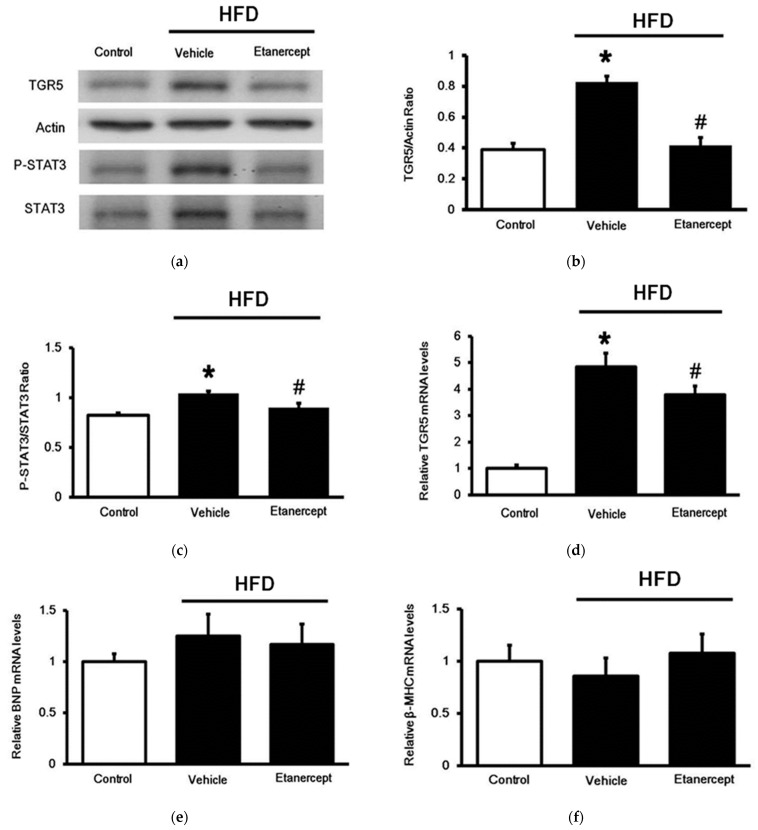
Expression of TGR5 in the hearts that were evaluated. The hearts were isolated from rats of three groups: (i) rats fed normal chow as control (control); (ii) HFD-fed rats treated with vehicle as the model control (vehicle); (iii) HFD-fed rats treated with etanercept (HFD + etanerceptf). Additionally, STAT3 activation was determined based on the ratio of STAT3 and p-STAT3. (**a**) The representative blot shows the protein levels of TGR5 and beta-actin, in addition to p-STAT3 and STAT3 in the heart tissues. (**b**) Quantification of the changes in TGR5 expression (TGR5 relative to beta-actin) and (**c**) STAT3 activation (p-STAT3 relative to STAT3) was also compared. (**d**) The mRNA levels of TGR5 expression in each group are shown. (**e**) The mRNA levels of genes related to cardiac hypertrophy, including BNPs and (**f**) β-MHC, were also compared. The data in each column are shown as the mean ± SEM (*n* = 4). * *p* < 0.05 vs. the control group; # *p* < 0.05 vs. the vehicle-treated group.

As shown in Figure 3, TGR5 expression at either the protein (Figure 3a) or the mRNA level (Figure 3d) was increased in the hearts of the HFD-fed rats compared to that in the hearts of the normal rats. Notably, changes in cardiac TGR5 expression were less marked in the HFD-fed rats treated with etanercept than in those treated with the vehicle only. This finding suggests that etanercept may alleviate HFD-induced changes in terms of TGR5 expression. Additionally, as shown in Figure 3c, similar changes were also observed in the expression of cardiac STAT3. Changes in TGR5 expression seem to be associated with STAT3 activation (Figure 3b) [8]. However, the mRNA levels of the genes related to cardiac hypertrophy, including brain/B-type natriuretic peptides (BNPs) (Figure 3e) and β-myosin heavy chain (β-MHC) (Figure 3f), remained unchanged in the hearts of the HFD-fed rats. Therefore, induction of cardiac hypertrophy in the HFD-fed rats at this stage seems unlikely.

### 3.4. Changes of TGR5 Expression in Urinary Bladder of HFD-Fed Rats

**Figure 4:**
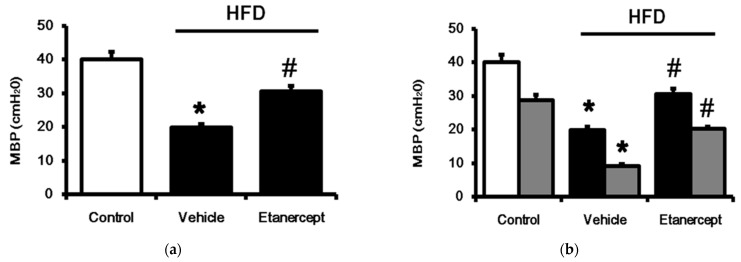

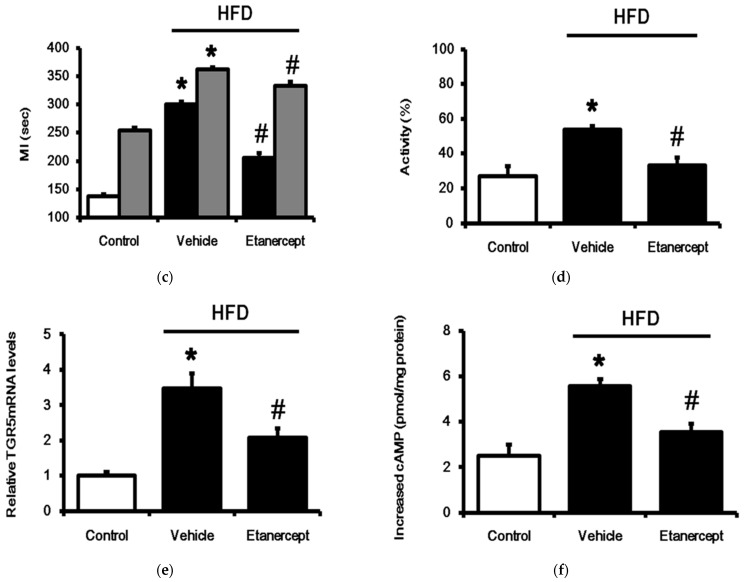
Changes in the urinary bladder of female rats. Female rats of three groups were used: (i) rats fed with normal chow (control); (ii) HFD-fed rats treated with vehicle (HFD); (iii) etanercept-treated rats that received HFD (HFD + etanercept). Basic contractility in each group, as seen from the cystometrogram, was compared (**a**). TGR5 activated by betulinic acid (50 mg/kg, ip) or not (vehicle) for changes in voiding contraction (**b**) and micturition frequency (**c**) among the three groups, as seen from the cystometrogram, were also compared. The voiding contraction decreased by betulinic acid, calculated as the ratio (%) of the non-treated contraction, was indicated as the activity of betulinic acid for a comparison among the three groups (**d**). The results in each column are indicated as the mean ± SEM (*n* = 8 per group). Additionally, changes in the mRNA levels of TGR5 expression in isolated urinary bladders of each group were compared (**e**). Increased cAMP levels by betulinic acid (5 µM) through TGR5 activation were also compared among the three groups (**f**). The results in each column are indicated as the mean ± SEM (*n* = 4 per group). * *p* < 0.05 vs. the control group; # *p* < 0.05 vs. the vehicle-treated group.

To understand whether or not the increased TGR5 expression is specific to the heart, we used female rats to investigate the changes in the urinary bladder, as described in a previous report [32]. Generally, a higher bladder pressure indicates lower bladder compliance. The maximum pressure significantly decreased in the HFD-fed rats, as shown in Figure 4a. Additionally, the micturition intervals in the HFD-fed rats were also significantly longer than those in the control rats. Notably, the blockade of TNF-α with etanercept reversed the urinary dysfunction in the HFD-fed female rats. Because LCA was less effective in urinary function [26], we applied another agonist of TGR5, betulinic acid, as described previously [33]. In the preliminary experiments, betulinic acid could induce the relaxation of the urinary bladder in normal rats that was abolished by pretreatment with triamterene at the dose effective to block TGR5 [34]. Therefore, the effects induced by betulinic acid were considered as the results of TGR5 activation. Betulinic acid (50 mg/kg, ip) may reduce voiding contraction and micturition frequency in HFD-fed rats. Notably, the relaxation induced by betulinic acid in the urinary bladder was more marked in the HFD-fed rats than in the normal rats (Figure 4b). Similarly, a delay of micturition intervals by betulinic acid was also observed in the same manner (Figure 4c). Blockade of TNF-α with etanercept markedly alleviated these changes in the urinary bladder. However, the relaxation induced by betulinic acid was more significant in the HFD-fed rats (Figure 4d). Additionally, the mRNA levels of TGR5 in the isolated urinary bladder were also determined. As shown in Figure 4e, the mRNA levels of TGR5 were markedly higher in the urinary bladder of the HFD-fed rats than in that of the normal rats, while etanercept reversed these changes in TGR5 expression. Moreover, an increase in cAMP levels caused by betulinic acid (5 µM) through TGR5 activation was similarly produced in the three groups (Figure 4f). Collectively, TGR5 expression is promoted in the urinary bladder of HFD-fed rats.

## 4. Discussion

In the present study, TGR5 expression in the heart or urinary bladder was noted to be increased in HFD-fed rats. This finding is consistent with the hyperglycemia-induced changes in the hearts of diabetic rats [9]. Insulin resistance is exacerbated by an increase in inflammation, along with a parallel increase in the activation of TGR5 [35], which may play a protective role in obese rats. TGR5 was identified in vivo, which may be targeted by bile acids [5] in both the healthy and diseased states [9]. TGR5 activation is beneficial to cardiac function [8] and MetS. TGR5 activation can ameliorate insulin resistance through the cAMP/PKA pathway in skeletal muscles [36]. However, at high concentrations, bile acids may stimulate cholinergic M2 receptors, which cause negative effects on myocardial contractility and heart rate [7]. In the present study, we demonstrated a novel view that TGR5 expression in the heart or urinary bladder is increased in HFD-fed rats.

First, we confirmed the cardiac functional response to TGR5 using the Langendorff apparatus. In the hearts isolated from normal rats, LCA enhanced cardiac contractility and decreased the heart rate due to TGR5 activation. TGR5 transduces signals through Gs protein-mediated cAMP accumulation and can modulate cardiac functions [1]. Moreover, the LCA-induced increase in contractility was more marked in the hearts isolated from HFD-fed rats than in hearts isolated from normal rats, indicating the increased sensitivity of TGR5 in the hearts of HFD-fed rats. However, spontaneous contractility was found to be reduced in the hearts isolated from HFD-fed rats compared to those isolated from normal rats. This result may be due to the reduced spontaneous contractility in HFD-fed rats. Moreover, we found that cardiac TGR5 expression truly increased in the hearts of HFD-fed rats at the protein and mRNA levels using Western blotting analysis and qPCR, respectively. Therefore, cardiac TGR5 expression was identified to be promoted in HFD-fed rats.

High-fat consumption is specifically known to be a causal factor in the development of cardiac damage [37]. The reductions in spontaneous contractility in HFD-fed rat hearts are consistent with this view. Cardiac damage is an inflammatory injury dependent on oxidative stress [38]. Additionally, a HFD induces insulin resistance and increases TNF-α expression. TNF-α promotes neutrophil-mediated tissue injury and amplifies inflammatory cascades by activating macrophages and other types of cells [39]. Functionally, TNF-α exerts a negative inotropic effect to inhibit myocardial contractility and lower blood pressure [40]. In the present study, the plasma levels of TNF-α and other cytokines markedly increased in HFD-fed rats. Moreover, we used etanercept at an effective dose to inhibit TNF-α in rats [23] as the negative control. The different changes in HFD-fed rats receiving etanercept may indicate the role of TNF-α. Notably, the changes in HFD-fed rat hearts were reversed by etanercept, as determined by a Langendorff assay. Therefore, the cardiac injury induced in HFD-fed rats seems to be associated with TNF-α, which is consistent with the findings of a previous report [41]. Moreover, in the current study, etanercept reversed cardiac TGR5 expression at both the protein and mRNA levels in HFD-fed rats.

STAT3 is a cytoplasmic transcription factor that transmits extracellular signals to the nucleus [42]. Activated STAT3 in the nucleus binds to specific DNA promoter sequences to regulate gene expression [43]. In the current study, cardiac TGR5 expression was promoted in parallel with STAT3 activation. Interestingly, etanercept also inhibited the activation of STAT3 in HFD-fed rat hearts. TNF-α inhibitors, etanercept, and adalimumab can downregulate p-STAT3 expression in human Th17-polarized cells [37]. STAT3 activation provides an important link between inflammation and cardiac fibrosis [38]. STAT3 accumulation in the nucleus can increase the expression of pro-inflammatory cytokine IL-6, which is involved in the pathogenesis of various chronic inflammatory diseases [39]. In the current study, plasma TNF-α and IL-6 levels that increased in HFD-fed rats were also found to be reduced by etanercept. Therefore, etanercept-mediated inhibition of TNF-α may result in downregulation of the IL-6/JAK/STAT3 pathway in HFD-fed rats. Additionally, STAT3 accumulation in the nucleus can also induce the expression of IL-6 and other proinflammatory genes [44]. Moreover, TNF-α can induce cardiac apoptosis, which is also involved in ventricular remodeling [41]. Therefore, the changes in TGR5 expression need to be investigated further.

Inflammation increases STAT3 activation, which contributes to the pathophysiology of tissue injury [45]. STAT3 activation and an increase in the ratio of phosphorylated STAT3 (p-STAT3) to STAT3 may promote nuclear translocation. Moreover, STAT3 was phosphorylated at Y705 and S727 in cells during cytokine-induced STAT3 activation [46]. Therefore, in the current study, we focused on changes in the ratio of p-STAT3 to STAT3, which is indicative of STAT3 activation. Interestingly, STAT3 activation was enhanced, along with the promotion of TGR5 expression in the heart. Our data also demonstrated that the increased ratio of p-STAT3 to STAT3 was reversed by etanercept in the hearts of HFD-fed rats. Mediation of STAT3 activation in terms of increased expression of cardiac TGR5 in HFD-fed rats can thus be considered.

Additionally, to understand whether or not the increased TGR5 expression was specific to the heart, we used female rats to investigate the changes in the urinary bladder, as described previously [29]. The changes were the same as those observed in the hearts. TGR5 expression in the urinary bladder was reduced in HFD-fed rats, as shown in a cystometrogram. This effect was reversed by etanercept at an effective dose to inhibit TNF-α in rats [22], indicating the role of cytokines in changes in the urinary bladder of HFD-fed rats. The high expression of TGR5 in urinary bladders was also a characteristic feature in these rats. We used betulinic acid to replace LCA for the activation of TGR5 in the urinary bladder. Betulinic acid is a natural triterpene that has been demonstrated to activate TGR5 [33]. Notably, relaxation in the urinary bladder by betulinic acid was more marked in HFD-fed rats than in normal rats, as determined from a cystometrogram. This view was supported by the increased mRNA levels of TGR5 in urinary bladders isolated from HFD-fed rats. Moreover, TGR5 is a member of the family of GPCRs that may increase cAMP levels [1]. We found that betulinic acid induced an increase in cAMP more markedly in the urinary bladders isolated from HFD-fed rats than in those isolated from normal rats. Therefore, TGR5 expression is increased in the urinary bladder during metabolic disorders. An increase in TGR5 expression could be a compensatory response against the lipotoxicity observed in HFD-mediated damage. However, this hypothesis needs further investigation in the future. The main limitation of this study is that the effect of etanercept in normal rats was not compared. The relationships between etanercept and TGR5 expression in a MetS model require further investigation.

## 5. Conclusions

We found that TGR5 expression is elevated in the heart or urinary bladder of HFD-fed rats. Notably, etanercept is effective in ameliorating inflammation and decreases TGR5 expression in HFD-induced obese rats. These results have implications for dysfunction in the heart or urinary bladder, particularly the association between inflammatory cytokines and TGR5 activation, which provides the benefit of reversing the dysfunction. The development of tissue-specific drugs that target TGR5 expression could provide benefits for assessing interventions in metabolic disease.

## Data Availability

Not applicable.
